# A Disease Resistance Elicitor Laminarin Enhances Tea Defense against a Piercing Herbivore *Empoasca* (*Matsumurasca*) *onukii* Matsuda

**DOI:** 10.1038/s41598-018-37424-7

**Published:** 2019-01-28

**Authors:** Zhaojun Xin, Xiaoming Cai, Shenglong Chen, Zongxiu Luo, Lei Bian, Zhaoqun Li, Lingang Ge, Zongmao Chen

**Affiliations:** 1 Tea Research Institute, Chinese Academy of Agricultural Sciences, Hangzhou, 310008 China; 2 Key Laboratory of Tea Biology and Resources Utilization, Ministry of Agriculture, Hangzhou, 310008 China

## Abstract

The tea plant (*Camellia sinensis*) suffers heavily from a harmful piercing pest, the tea green leafhopper (TLH) *Empoasca* (*Matsumurasca*) *onukii* Matsuda. In the present study, we studied the effect of an efficient elicitor of plant disease resistance, the β-1,3-glucan laminarin, on the induced defense against TLH in tea plants. Defense responses elicited by laminarin in tea include the activation of mitogen-activated protein kinases and WRKY, the burst of H_2_O_2_, salicylic acid, and abscisic acid, and the accumulation of direct-defense chemicals (including chitinase, phenylalanine ammonia lyase, callose, polyphenol oxidase, and flavonol synthase), as well as the production of volatile compounds. The laminarin-treated tea plants reduced the performance of TLH and enhanced the attractiveness to the egg parasitoid wasp of TLH, *Stethynium empoascae* Subba Rao. In the field experiment, laminarin application effectively reduced the number of TLH by attracting parasitoids. These results suggest that laminarin can induce protection against TLH by regulating signaling pathways in tea plant. Our study also proposes an environment friendly strategy for the integrated management of an economically important piercing pest.

## Introduction

As sessile organisms, plant populations are subject to attack by enormous diversity of herbivorous insects in their life cycles. Thus, the plants have developed an array of induced defense mechanisms when they are infested with pests^1^. The induced plant defense mechanism is usually associated with mitogen-activated protein kinase (MAPK) cascades^2^ and signaling pathways mediated by phytohormones, such as the jasmonic acid (JA), salicylic acid (SA), abscisic acid (ABA), and ethylene (ET), which can activate signal transduction cascades that finally trigger plant defense reactions. These defense reactions include upregulated defense-related gene expression, and accumulated levels of defense-related compounds, such as lectins, chitinases, proteinase inhibitors, nicotine, phenylalanine ammonia lyase (PAL) and polyphenol oxidases (PPOs)^3,4^, as well as the emission of herbivore-induced plant volatiles (HIPVs) that can repel insects or attract their natural enemies^5,6^.

Interestingly, plants use different defense pathways to respond to insects with different feeding modes^7^. For example, JA pathway can effectively induces defense response against chewing herbivores, such as caterpillars and beetles which cause extensive tissue damages^8^. By contrast, SA and ABA pathways play significant roles in plants defense against several piercing/sucking herbivores, such as aphids, planthoppers, mites, and whiteflies, whose stylet penetration caused weak wound like intercellular fungal hyphal growth^9,10^.

To date, accumulating evidences have indicated that plant defense mechanisms can be activated by various chemical elicitor types, including (i) naturally occurring phytohormones, such as JA, SA, ABA, and ET^1,11,12^; (ii) the other elicitors exist in plants, for example, green leaf volatiles or terpene compounds^6^; (iii) the synthetic elicitors not exist in plants, e.g., 3,5-dichloroanthranilic acid and 2,4-dichlorophenoxyacetic acid (2,4-D)^13,14^. These chemical elicitors are not directly toxic to pests but capable of inducing defense-related signaling pathways in plants and elicit extensive herbivore-defense properties^1,13,14^. Therefore, chemical elicitors may be deployed as a novel strategy to strengthen the biological control of harmful insects^15,16^.

The tea plant, *Camellia sinensis* (L.) O. Kuntze, is an important cash crop in the Asian countries, such as China, India, and Sri Lanka. Tender tea buds and leaves are usually plucked to produce high-grade tea as beverage. The dietary antioxidants (such as polyphenols) contained in the beverage are beneficial to the human health^17^. Like other crops, *C. sinensis* suffers heavily from many herbivorous insects in their life cycles. The tea green leafhopper (TLH), *Empoasca* (*Matsumurasca*) *onukii* Matsuda (Hemiptera: Cicadellidae), an extremely harmful piercing pest with ten generations per year, is by far the most serious threat to tea plant cultivation^18^. Both nymphs and adults of *E. onukii* attack tea plants by using its piercing mouthparts (stylet), and ultimately results in the plant’s yellowing, browning, and drying^19^. The most common methods used to control TLH are regularly applying chemical insecticides. However, the excessive use of pesticides is hazardous for both the environment and the human health. Therefore, exploiting chemical elicitors is an efficient strategy to defend tea plants against TLH^16,20^.

The β-1,3-glucan laminarin^21^, a storage polysaccharide from the brown algae *Laminaria digitata*, was reported to be an efficient elicitor of disease resistance in various plant species including alfalfa^22^, rice^23^, tobacco^24^, and grapevine^25^. In grapevine, laminarin could induce cytosolic Ca^2+^ surge, oxidative burst, accumulate expression of pathogenesis-related genes, and effectively reduce the development of two pathogens *Botrytis cinerea* and *Plasmopara viticola*^25^. Recently, laminarin was reported to modulate the chloroplast antioxidant system to enhance tolerance to abiotic stress in *Arabidopsis*^26^. In maize, Sobhy *et al*.^27^ studied the effects of laminarin on the emissions of HIPVs and parasitoid attraction. Although the role of laminarin in inducing plant disease resistance has have been confirmed, its function on modulating plant defense against herbivores and the corresponding molecular mechanisms are remained largely unknown.

In the present study, we found that laminarin can effectively trigger tea early defense responses including the activation of MAPK and WRKY, and the enhancement SA, H_2_O_2_, ABA production. Moreover, laminarin increased the accumulation of several direct and indirect defense-related compounds, and thus enhanced tea defense against TLH under laboratory and field conditions. Our results revealed the defense mechanism of laminarin acts as an herbivore-resistance elicitor in tea plant and confirmed the effectiveness of laminarin in the integrated management of an economically important piercing pest.

## Results

### Effect of laminarin on signaling events in tea plants

**Figure 1:**
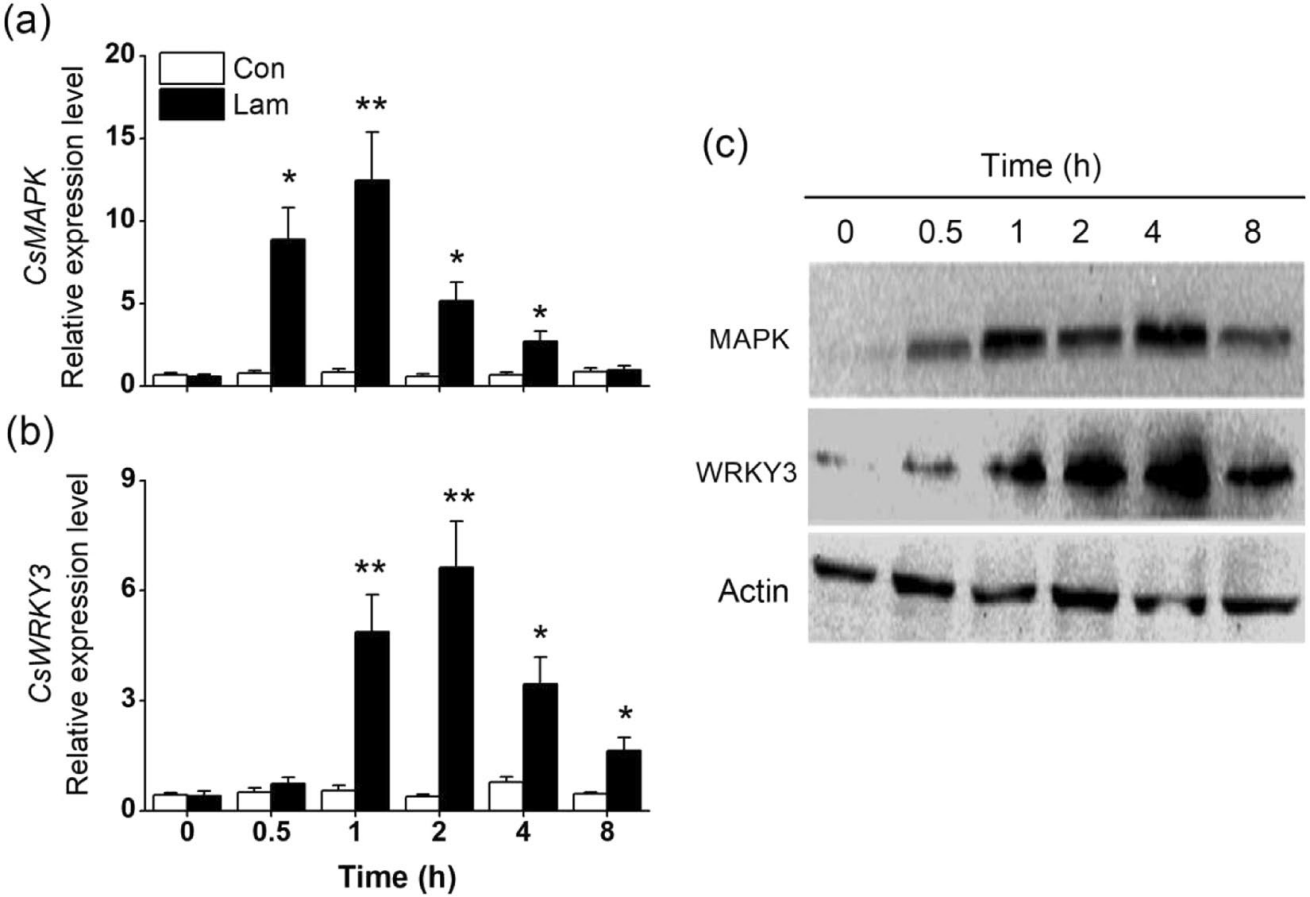
Laminarin regulates mitogen-activated protein kinase (CsMAPK) and CsWRKY3. Mean transcript levels (+SE, n = 5) of *CsMAPK* (a) and *CsWRKY3* (**b**) in leaves of tea plants treated with laminarin at a concentration of 200 mg L^−1^ (Lam), relative to controls (Con). (**c**) Western blot analysis of the accumulation of CsMAPK and CsWRKY3. The grouping of blots was cropped from different parts of the same gel. Asterisks indicate significant differences in transcript levels between treatments and controls at each time point (*P < 0.05; **P < 0.01; Student’s t-test).

MAPK cascades and WRKY transcription factors play broad and pivotal roles in triggering plant defense responses and early signaling mechanisms^2,28^. Thus, we investigated whether treatment with laminarin alters the expression of *CsMAPK* (the homologs of *NaWIPK* in *Nicotiana attenuata*) and *CsWRKY3*^29^ (the homolog of *NaWRKY6*) in *C. sinensis*. Transcript levels of the *CsMAPK* and *CsWRKY3* increased and peaked at 1–2 h after treatment (Fig. 1a,b). Similar with the gene expression results, western blot showed laminarin treatment also enhanced the accumulation of CsMAPK and CsWRKY3 proteins (Fig. 1c).

**Figure 2:**
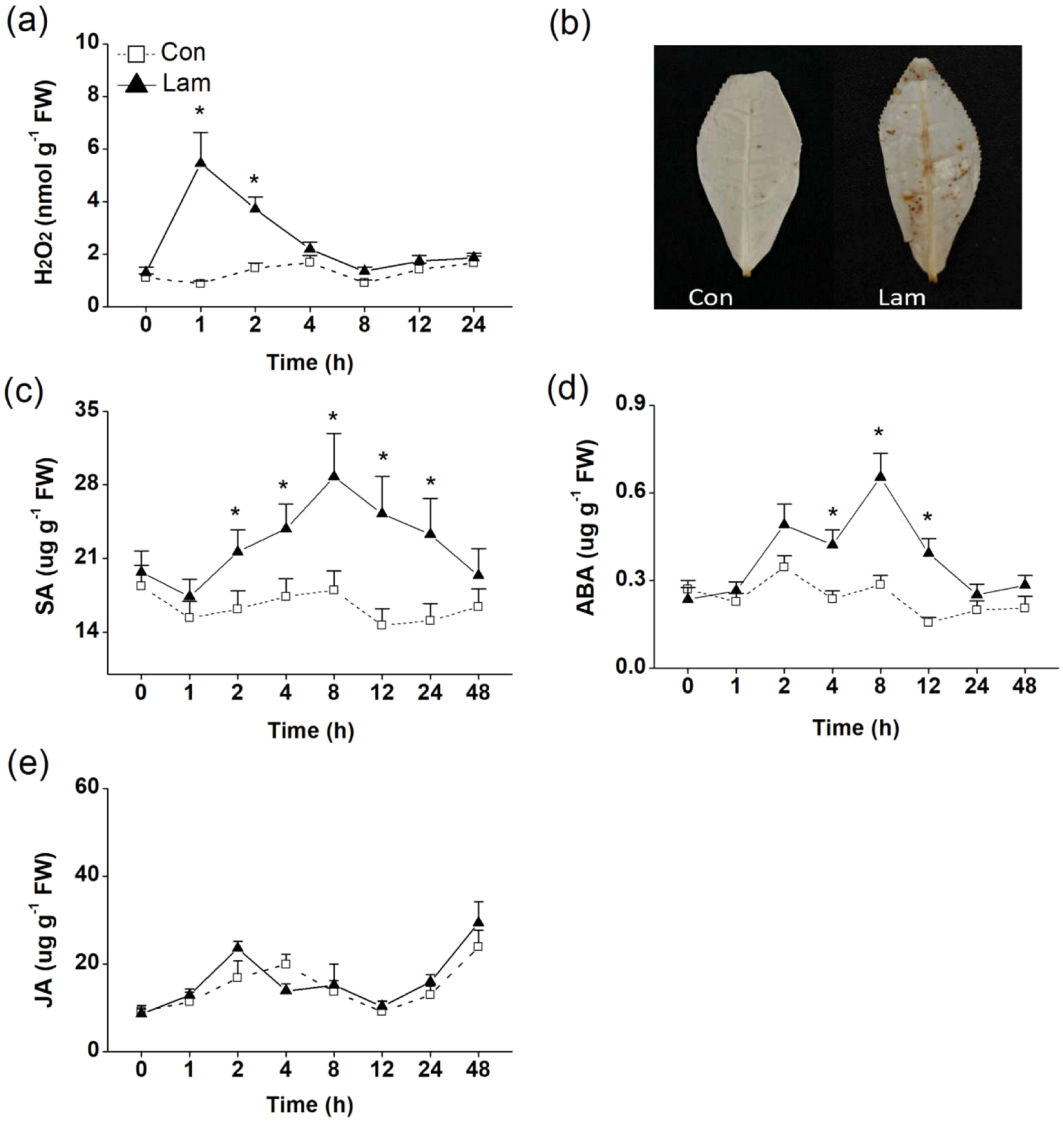
Mean levels of different defense-related signal molecules in tea plants. The level of H_2_O_2_ (**a**), SA (**c**), ABA (**d**) and JA (**e**) contents in control (Con) and laminarin-treated sets (Lam) were analyzed at different time intervals. (**b**) DAB staining analysis of H_2_O_2_ level in tea leaves treated with laminarin (Lam) or control (Con). The values represent means + standard error (SE) of five repeats. Asterisks indicate the significant differences between treatments and controls at each time point (*P < 0.05; **P < 0.01; Student’s t-test).

Three plant defense-related signal molecules, H_2_O_2_, SA and ABA can be induced by laminarin. The enhancement of H_2_O_2_ was first induced at 1 h and rapidly disappeared at 4 h (Fig. 2a). One hour after laminarin treatment, foliar tissue of tea leaves stained reddish-brown after DAB staining. Very little staining was detectable in control leaves (Fig. 2b). Both SA and ABA levels increased at 2–24 h in the leaves of laminarin-treated tea plants, and peaked at 8 h (Fig. 2c,d). The JA levels were not influenced by laminarin treatment (Fig. 2e). Consistently, the relative expression of *CsOPR3*, a key enzyme in the biosynthesis of JA^30^, and CsOPR3 protein was also not accumulated in tea leaves after laminarin treatment (Fig. S1).

### Effect of laminarin on the defense-related compounds

**Figure 3:**
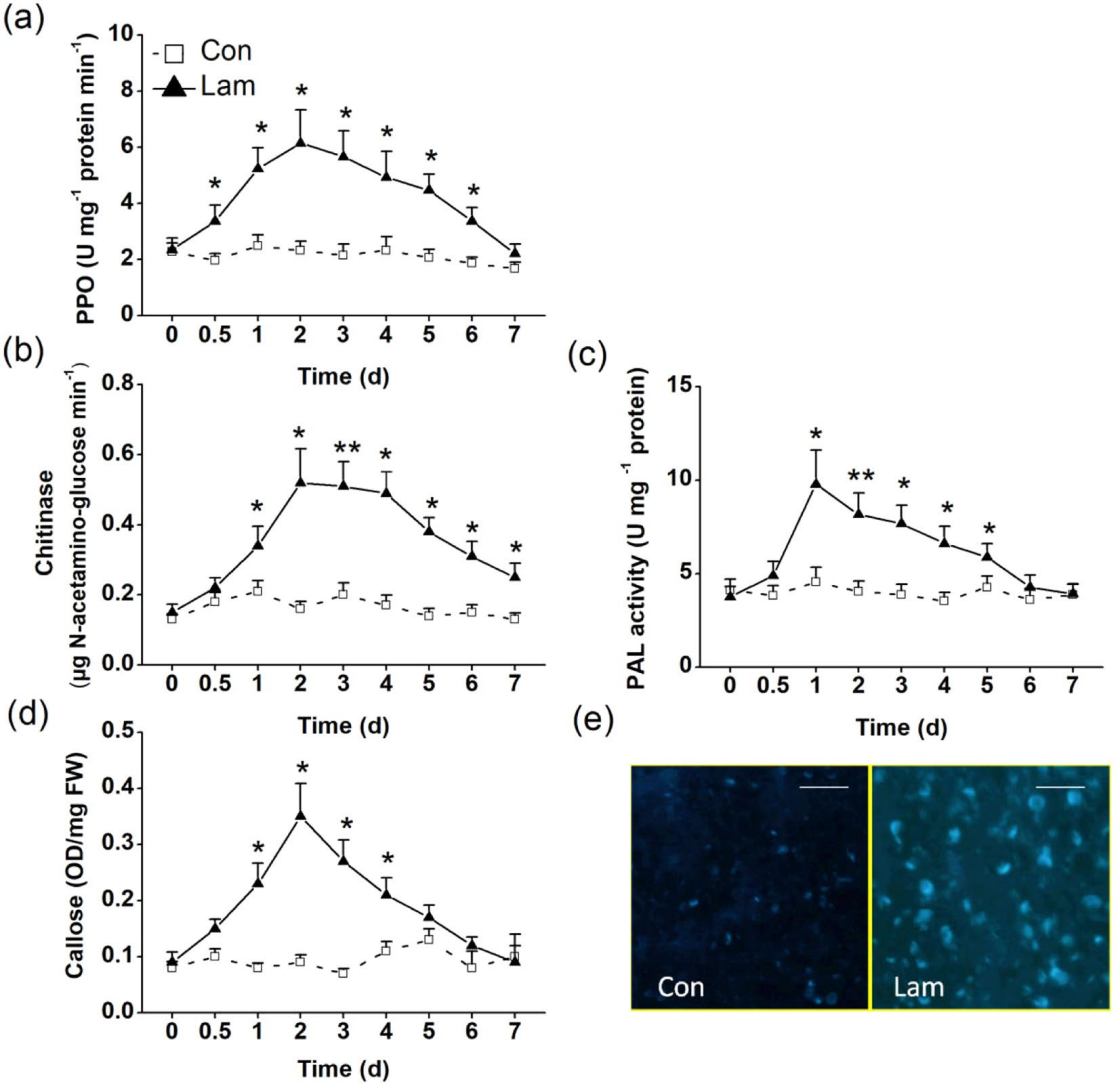
Mean activity levels of different defense-related compounds in tea plants. The activity of PPO (**a**), chitinase (**b**), and PAL (**c**) and the content of callose (**d**) in the control (Con) and laminarin-treated leaves (Lam) were analyzed at different time intervals. (**e**) Callose deposition in the control (Con) and laminarin-treated leaves (Lam). Leaflets were stained with aniline blue to detect callose. Representative microscope images are shown. Scale bars represent 50 μm. Values represent the mean + SE of five repeats. Asterisks indicate the significant differences between treatments and controls at each time point (*P < 0.05; **P < 0.01; Student’s *t*-test).

**Figure 4:**
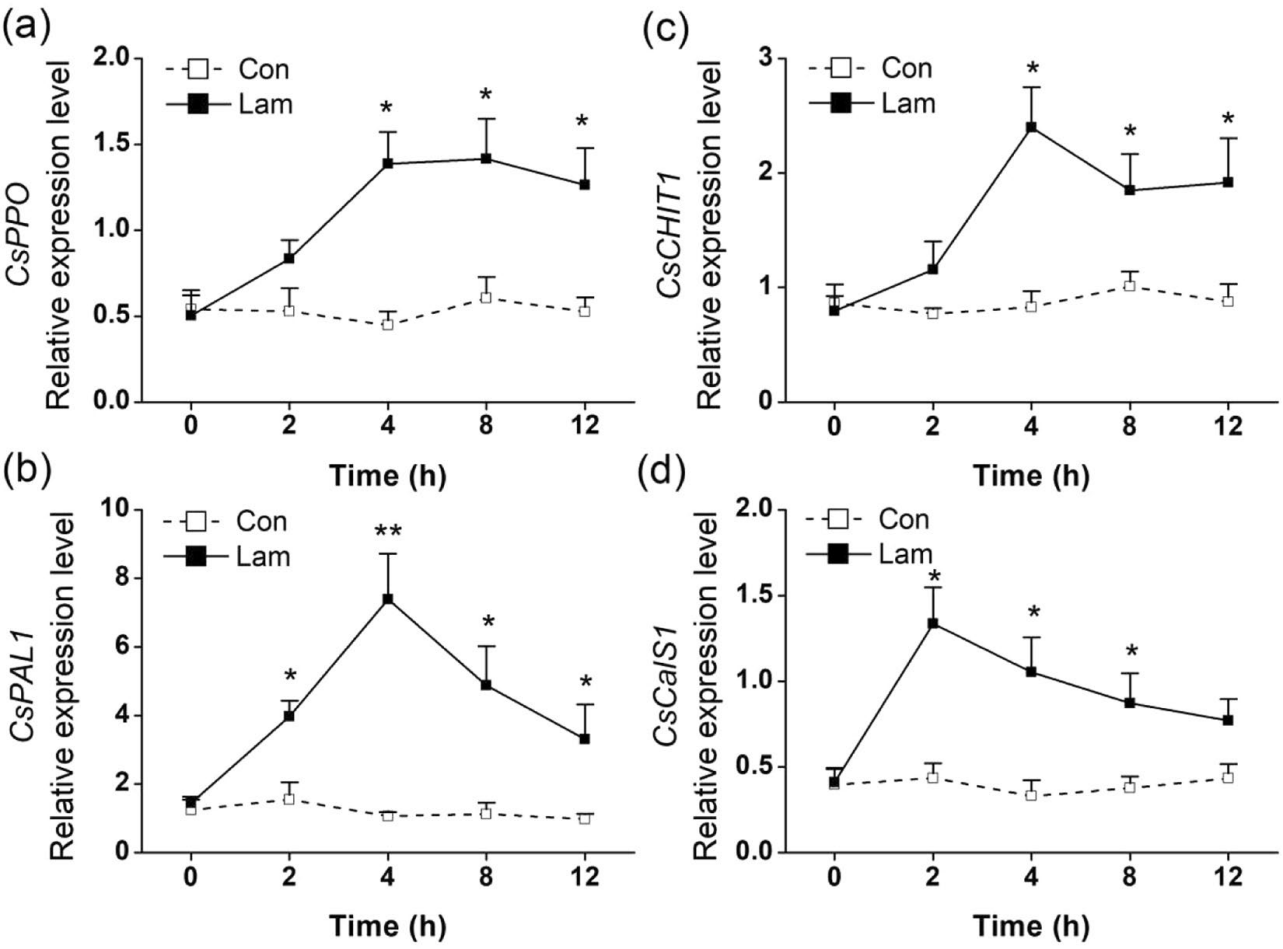
Transcript accumulation of defense genes in tea leaves after elicitation by laminarin. Mean transcript levels (+SE, n = 5) of *CsPPO* (a), *CsPAL1* (**b**), *CsCHIT1* (**c**) and *CsCalS1* (**d**) in leaves of tea plants treated with laminarin (Lam), relative to controls (Con). Asterisks indicate significant differences in transcript levels between treatments and controls at each time point (*P < 0.05; **P < 0.01; Student’s *t*-test).

**Figure 5:**
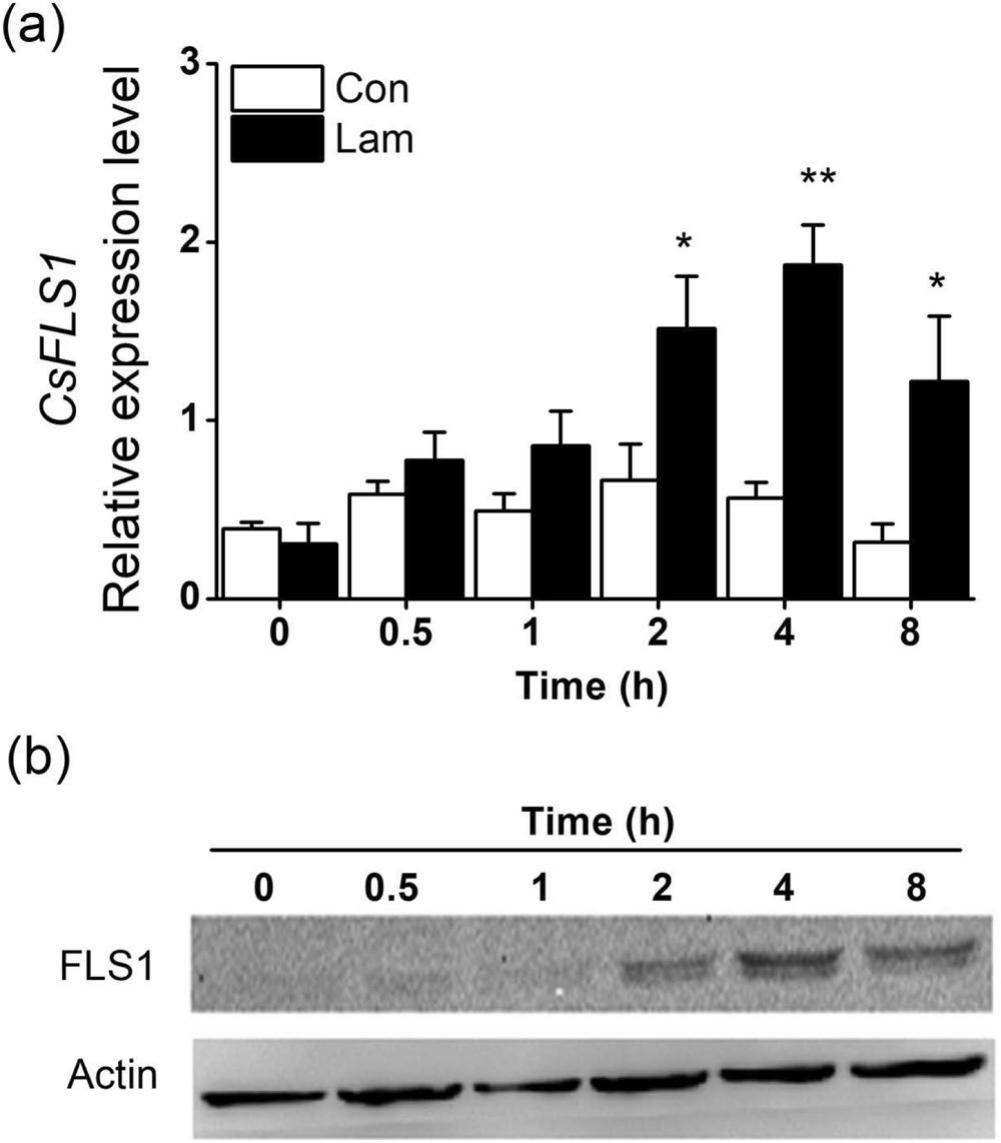
Laminarin regulates the biosynthesis of flavonol synthase (FLS). (**a**) Mean transcript levels (+SE, n = 5) of *CsFLS1* in leaves of tea plants treated with laminarin at a concentration of 200 mg L^−1^ (Lam), relative to controls (Con). (**b**) Western blot analysis of the accumulation of CsFLS1. The grouping of blots was cropped from different parts of the different gel. Asterisks indicate significant differences in transcript levels between treatments and controls at each time point (*P < 0.05; **P < 0.01; Student’s *t*-test).

Biochemical analysis revealed that the PPO activity in the laminarin-treated plants increased significantly on the 0.5 d, peaked at 2 d, and then decreased gradually (Fig. 3a). Laminarin treatment also enhanced chitinase activity, reached maximum at the second day, and maintained such induction to the seventh day (Fig. 3b). The PAL activity increased and peaked sharply after 1 d of laminarin treatment, and maintained up to the fifth day (Fig. 3c). Laminarin also enhanced the callose content 1 d after the treatment, peaked at 2 d, and then decreased gradually (Fig. 3d). Consistent with the result of callose content, the laminarin-treated leaflets exhibited an obvious induction of callose deposition compared with the control plants (Fig. 3e). In addition, laminarin treatment within 12 h also upregulated the relative expression levels of four genes, *CsPPO, CsCHIT1, CsPAL1*, and *CsCalS1*, putative encoding PPO, chitinase, PAL and callose synthetase (Fig. 4a–d). We also found laminarin has a positively effect on a flavonol synthase (FLS). Both the transcript level of *CsFLS1* and CsFLS1 protein were accumulated in tea leaves after laminarin treatment (Fig. 5a,b).

### Effects of laminarin on tea volatile emissions

**Figure 6:**
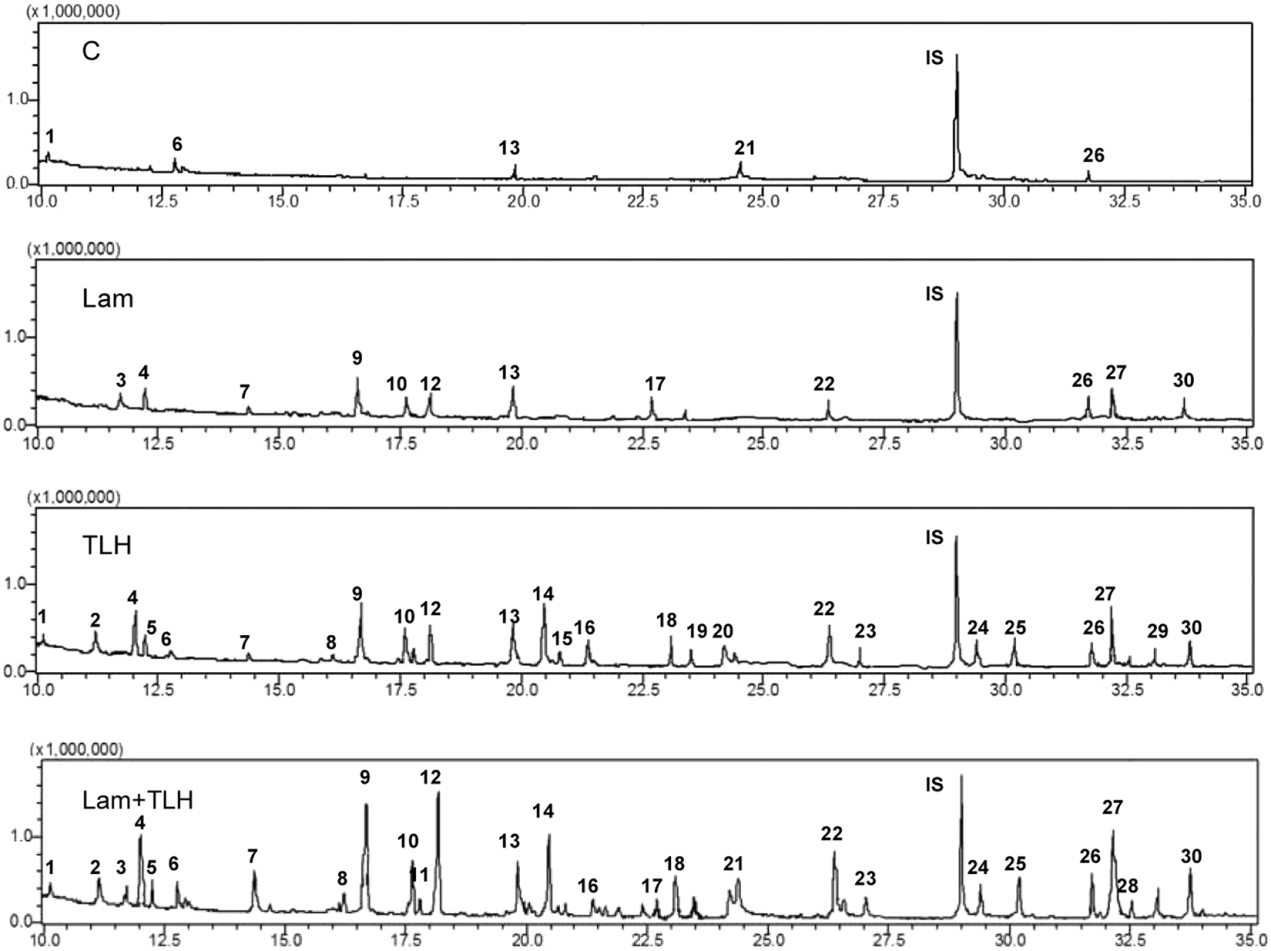
Volatile compounds identified from the headspace collections of the control (C), laminarin-treated (Lam), TLH-infested (TLH) and laminarin-treated, TLH-infested (Lam + TG) tea plants. Numbers represent the chemicals corresponding to those written in Table 1.

We then measured the effects of 200 mg L^−1^ laminarin on the emissions of tea constitutive and TLH-induced volatiles. Five compounds with small quantities were detected in the control plants (Table 1; Fig. 6). Laminarin increased the total amount of tea volatiles significantly relative to those of the controls. Twelve volatile compounds were detected in the headspace of laminarin-induced plants (Table 1; Fig. 6). When plants were treated with TLH for 24 h, 28 compounds were collected (Table 1; Fig. 6). Treatment with laminarin also enhanced the emission of TLH-induced tea volatiles (Fig. 6; Table 1). The amounts of 12 compounds including 4 GLVs ((*E)*-2-hexenal, (*Z*)-3-hexenol, (*Z*)-3-hexenyl acetate, and (*Z*)-3-hexenyl butyrate), 6 terpenes ((*Z*)-β-ocimene, (*E*)-β-ocimene, Linalool, (*E*)-caryophyllene, (*E,E*)-α-farnesene, and (*E*)-nerolidol), indole, and 1 unknown compounds (unknown 4) were significantly higher in the Lam + TLH-treated plants than in the TLH-treated plants (Table 1; Fig. 6).

**Table 1:** Comparison of volatile compounds (mean ± SE; *n* = 6) emitted from plants infested with TLH (TLH), treated with laminarin (Lam), the combination of both treatments (Lam + TLH), and controls (C).

No	Compound	C	Lam	TLH	Lam + TLH
1	(*Z*)-3-hexenal^a^	0.02 ± 0.01 b	ND	0.51 ± 0.11a	0.43 ± 0.06 a
2	Unknown 1	ND	ND	0.49 ± 0.08 a	0.78 ± 0.18 a
3	(*E*)-2-hexenal^b^	ND	0.11 ± 0.03 a	ND	0.20 ± 0.07 a
4	(*Z*)-3-hexenol^b^	ND	0.31 ± 0.08 c	0.90 ± 0.17 b	3.23 ± 0.40 a
5	Unknown 2	ND	ND	0.38 ± 0.10 a	0.47 ± 0.12 a
6	Unknown 3	0.10 ± 0.04 b	ND	0.43 ± 0.11 a	0.61 ± 0.17 a
7	Unknown 4	ND	0.03 ± 0.01 b	0.05 ± 0.01 b	0.86 ± 0.22 a
8	Unknown 5	ND	ND	0.06 ± 0.02a	0.11 ± 0. 03 a
9	(*Z*)-3-hexenyl acetate^a^	ND	0.73 ± 0.15 c	2.87 ± 0.61 b	5.62 ± 0.85 a
10	(*Z*)-β-ocimene^b^	ND	0.33 ± 0.08 b	0.55 ± 0.13 b	1.22 ± 0.28 a
11	Benzyl alcohol^c^	ND	ND	0.15 ± 0.04 a	0.22 ± 0.05 a
12	(*E*)-β-ocimene^b^	ND	0.25 ± 0.06 c	1.16 ± 0.22 b	6.76 ± 1.02 a
13	Linalool^d^	0.11 ± 0.03 d	0.49 ± 0.09 c	1.37 ± 0.38 b	2.88 ± 0.61 a
14	Phenylethyl alcohol^d^ + DMNT^e^	ND	ND	3.63 ± 0.52 a	4.35 ± 0.82 a
15	Unknown 6	ND	ND	0.13 ± 0.05 a	0.20 ± 0.08 a
16	Benzyl nitrile^d^	ND	ND	0.35 ± 0.08 a	0.41 ± 0.07 a
17	(*Z*)-3-hexenyl butyrate^a^	ND	0.53 ± 0.12 a	ND	0.72 ± 0.16 a
18	(*E*)-2-hexenyl butyrate^a^	ND	ND	0.51 ± 0.13 a	0.88 ± 0.19 a
19	Methyl salicylate^a^	ND	ND	0.22 ± 0.05 a	0.31 ± 0.08 a
20	(*Z*)-3-hexenyl-2-methyl butyrate^f^	ND	ND	0.25 ± 0.07 a	0.35 ± 0.09 a
21	Unknown 7	ND	ND	0.26 ± 0.06 a	0.32 ± 0.07 a
22	Indole^f^	ND	0.28 ± 0.07 c	1.55 ± 0.29 b	2.62 ± 0.39 a
23	Phenyl ethane (1-nitro-2-)^g^	ND	ND	0.31 ± 0.07 a	0.41 ± 0.09 a
24	(Z)-3-hexenyl hexanoate^f^	ND	ND	0.60 ± 0.18 a	0.39 ± 0.12 a
25	Unknown 8	ND	ND	0.93 ± 0.30 a	1.11 ± 0.25 a
26	(*E*)-caryophyllene^c^	0.03 ± 0.01 c	0.16 ± 0.03 bc	0.28 ± 0.06 b	0.59 ± 0.17 a
27	(*E*,*E*)-α-farnesene^h^	ND	0.36 ± 0.07 b	0.79 ± 0.17 b	2.36 ± 0.53 a
28	Unknown 9	ND	ND	0.09 ± 0.03 a	0.11 ± 0.03 a
29	Unknown 10	ND	ND	0.15 ± 0.04 a	0.21 ± 0.06 a
30	(*E*)-nerolidol^h^	ND	0.39 ± 0.10 b	0.35 ± 0.08 b	0.79 ± 0. 22 a
Total		0.26 ± 0.06 d	3.97 ± 0.89 c	19.32 ± 3.81 b	39.52 ± 6.52 a

The amounts of individual volatile compounds were calculated by comparing their peak areas relative to those of the corresponding internal standards. Letters indicate the significant differences between treatments (P < 0.05; Duncan’s multiple-range test). The names of the compounds followed by different letters indicate different methods for confirming identities: a–g comparison of retention times and mass spectra with those of authentic standards as follows: ^a^Roth; ^b^Fluka; ^c^TCI; ^d^Acros; ^e^gift from Taro Maeda; ^f^Sigma-Aldrich; ^g^comparison of Kovats Indices (KI) on DB-5; ^h^Pherotech. ND: not detected.

### Laminarin activates direct and indirect resistance in the laboratory

**Figure 7:**
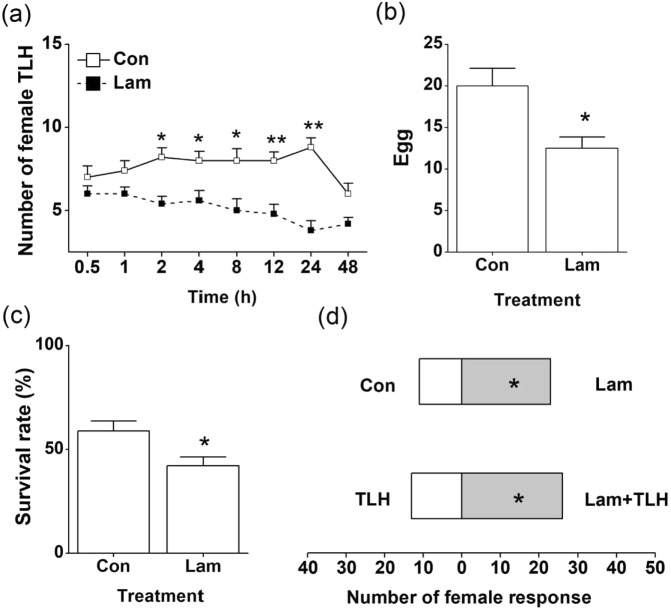
Effect of laminarin (Lam) treatment on tea plants’ direct and indirect resistance to TLH. (**a**) The mean number of TLH female adults (+SE, n = 6) on plants treated with laminarin vs. control plants, 0.5–48 h after exposure. (**b**) Mean percentage (+SE, n = 6) of TLH eggs per plant on pairs of plants, 72 h after the release of TLH. (**c**) Mean percentage (+SE, n = 6) of survival rate of TLH female adults per plant on control and laminarin-treated plants. (**d**) Behavioral responses of TLH egg parasitoid *S. empoascae* female adults to different plant volatiles released from pairs of odors: Control plants (Con) versus laminarin-treated plants (Lam); TLH-infested plants (TLH) versus both laminarin and TLH-treated plants (Lam + TLH). The values represent means + standard error (SE) of six repeats. Asterisks indicate significant differences between members of a pair (*P < 0.05; **P < 0.01; Student’s *t*-test).

Since laminarin mediates induced resistance-related signaling pathways and compounds associated with the defense against piercing insects, we hypothesized that laminarin likely protect tea plants against TLH. When TLH female adults were placed between two different treated plants, the pests were found more frequently on control plants than on the plants treated with laminarin (Fig. 7a). Similarly, TLH female adults laid significantly less eggs on laminarin-treated plants than on control plants (Fig. 7b). Moreover, TLH nymphs fed on the control plants achieved higher survival rates than those fed on laminarin-treated plants (Fig. 7c). Females of the TLH parasitoid *S. empoascae* were mostly attracted to odors of plants treated with laminarin, and preferred to choose the odors of Lam + TLH-treated plants more than the odors of TLH-infested plants (Fig. 7d).

### Laminarin enhances the protection of tea plants against TLH in the field

**Figure 8:**
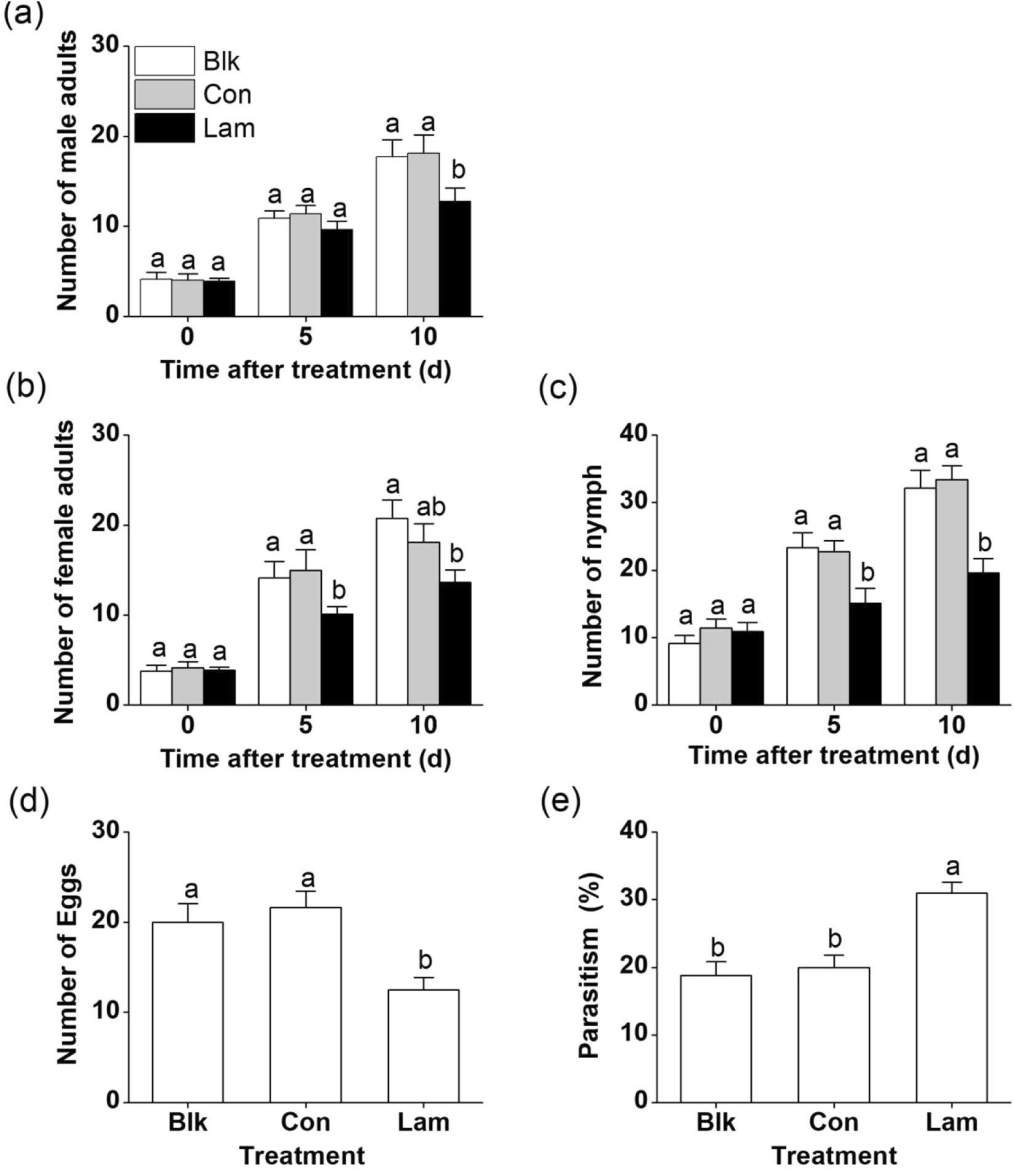
Effect of laminarin treatment on TLH populations in the field. (**a**–**c**) Mean number (+SE, n = 3) TLH male adults (**a**), female adults (**b**) and nymphs (**c**). 0, 5 and 10 days after plants were sprayed with laminarin at a concentration of 200 mg L^−1^ (Lam), or with buffer only (Con), relative to the Blank (Blk). (**d**,**e**) Mean number (+SE, n = 3) of TLH eggs (**d**) and parasitism of TLH by *S. empoascae* female adults (**e**), 10 days after plants were sprayed with laminarin at a concentration of 200 mgL^−1^ (Lam), or with buffer only (Con), relative to those of the Blank. Letters indicate significant differences between treatments or lines (*P* < 0.05, Duncan’s multiple-range test).

In the field, no obvious difference was found in the survey data collection between the blank and control plots (Fig. 8). However, the densities of female, male adults, nymphs, and eggs of TLH, were observed to be lower on laminarin-treated plots than on control or blank plots (Fig. 8a–d). Laminarin application strongly enhanced the parasitism of TLH eggs by the egg parasitoid *S. empoascae* (Fig. 8e). When tea plants were treated with laminarin, the parasitism of TLH eggs by *S. empoascae* on the plants was 55% higher than that on the control plants (Fig. 8e). The density of spiders was not increased by laminarin treatment (Fig. S2).

## Discussion

Applying elicitors to strengthen plants’ induced defense against harmful insects can be deployed as a novel biological control strategy^16,20^. In the present study, we provided evidences supporting foliar application a plant disease resistance elicitor, laminarin, can supply tea plants protection against the sap-sucking herbivore TLH in laboratory and field, and the efficacy of laminarin relied on the activation of tea induced defense responses.

Activation of MAPK cascades and WRKY transcription factors are early events in the mediation of plant resistance to pathogens/insects and the biosynthesis of defense-related phytohormones, such as JA, SA, and ET^31–33^. Generally, MAPK and WRKY play central roles in diverse physiological functions, through cross talk, both synergistic and antagonistic interactions^34,35^. In plants, many MAPK and WRKY modules participate in the perception of various biotic or abiotic stimuli. In tobacco, salicylic acid-induced protein kinase (SIPK) and wound-induced protein kinase (WIPK) are two MAPKs activated in response to various elicitors, including cryptogein and oligogalacturonides^36,37^. In rice, the synthetic compound 2,4-D enhanced the transcript of three MAPK genes (*OsMPK3, OsMPK6* and *OsMEK3*) and one WRKY gene (*OsWRKY53*)^14^. In this study, laminarin-treated tea plants showed a fast accumulation of *CsMAPK* (Fig. 1), the homolog of *NaWIPK* which positively regulated the biosynthesis of JA and SA in *N. attenuata*^31^, with higher levels of SA but not JA (Figs 2 and S1). A contrary phenomenon was found in 2,4-D-treated rice, in which the induction of MAPKs resulted in higher levels of JA, but reduced the levels of SA^14^. This suggests that the function of MAPK in modulating the JA or SA burst may differ among plant species or chemical elicitors. In addition, JA level was not induced by laminarin treatment in this study, one possible explanation maybe an antagonistic effect of the SA production (activated by MAPK) on the biosynthesis of JA^38^. These hypotheses need to be investigated further.

Plant signaling molecules (such as JA, SA, H_2_O_2_, and ABA) play distinct but overlapping roles in regulating the defense response, growth, and development of plants^1,12^. Application of laminarin to tea leaves enhanced the H_2_O_2_, SA, ABA production (Fig. 2). SA and H_2_O_2_ are ubiquitously occurring signaling molecules in plant defense, often produced after pathogen recognition^39,40^. They play vital roles in activating defense genes, establishing systemic acquired resistance (SAR), and inducing hypersensitive response (HR)^41^. H_2_O_2_ accumulation is the earliest detectable cytological responses in laminarin-treated tea leaves in the present study (Fig. 2a,b). SA level was also induced in laminarin-treated tea leaves (Fig. 2c). In addition, H_2_O_2_ and SA were reported to function in plant resistance to piercing herbivores^39,42^. SAR responses induced by SA and H_2_O_2_ could affect the performance of piercing herbivores in some plants^43^. For example, in rice, the high H_2_O_2_ and SA levels were accompanied with the enhanced resistance against an important piercing pest of rice, the brown planthopper (BPH), *Niaparvata lugens*^39^. Our results reconfirmed the overlapping roles of H_2_O_2_ and SA in the defense against pathogen and herbivores.

Interestingly, we found for the first time in this study that laminarin enhanced the level of ABA production in tea plants (Fig. 2d), indicating the activation of the ABA-mediated signaling pathway. ABA has been proved to enhance the antioxidant capacity when plants suffer stresses^44^. In some plant–pathogen interactions, ABA possesses the capability of enhancing the synthesis of callose, which produces an effective defense response against the attempted penetration of pathogens^45^. Hao *et al*.^10^ found that callose deposition could be induced by BPH infestation in the sieve tubes where the stylet was inserted in rice. Such callose plugging hindered the further feeding of BPH to rice. Recently, Liu *et al*.^12^ reported that exogenous ABA treatment significantly enhanced rice resistance against BPH. This resistance attributed to the increased callose content in rice. In this study, the laminarin-treated tea leaves exhibited a markedly induction in callose deposition compared with the control plants (Fig. 3d,e). Given the above–mentioned studies, we speculate that the augmented ABA (Fig. 2d) and callose deposition (Fig. 3d,e) attributed to the laminarin-induced tea plant’s resistance against TLH.

Besides the increased callose production, the accumulations of chitinase, PAL, and PPO, as well as the transcript levels of their putative encoding gene, were also observed in laminarin-treated plants (Figs 3 and 4). Both PPOs and chitinases are vital defense compounds existing widely in tea plants at large amounts. Chitinase participates in plant defense by hydrolyzing insect/fungal cell wall components^46^. The enzyme also amplifies the plant defense by releasing chitin fragments from the insect/pathogen cell walls^47^. PPOs can directly affect the feeding, fecundity, and survival rate of the invaders^48^. Laminarin treatment also increases the activity of PAL (Fig. 3c), a key enzyme of the phenylpropanoid pathway in plant development. PAL can catalyse the deamination of phenylalanine, resulting in the production of defense-related secondary metabolites, such as flavonoids, flavonols, anthocyanins, and SA^49^. We also found a FLS protein was accumulated in laminarin-treated tea leaves (Fig. 5). FLS protein, which is synthesized from chalcone via the phenylpropanoid and flavonoid pathways, could function in plant defense against insects^50^. In another report, laminarin treatment also enhances the biosynthesis of lignin^51^, which can prevent piercing/sucking insects from penetrating leaf tissues^52^. Therefore, we speculate that the laminarin-induced direct resistance against TLH (Fig. 7a–c) is correlated with the elevated activities of the above–mentioned defense-related compounds.

Attracting natural enemies by manipulating the release of plant volatiles using chemical elicitors is an effective biological strategy to control pests^15^. In rice, application of JA, MeJA, or 2,4-D enhanced the emission of volatiles and boosted the attraction of the egg parasitoid of BPH^14,53,54^. Similarly, benzo (1,2,3) thiadiazole-7-carbothioic acid S-methyl ester (BTH) promoted the cotton plants to release of a large number of homoterpenes and significantly increased the parasitoids attraction^15^. In the present study, the laminarin-treated tea plants increased the total amounts of constitutive and TLH-induced tea volatiles (Fig. 6; Table 1). The increased volatiles enhanced the attraction to the egg parasitoid *S. empoascae* (Fig. 7d). Interestingly, the maize plants treated with laminarin reduced HIPVs emissions, but still enhanced the attractiveness of three important parasitic wasps^27^, which indicates the functional diversification of laminarin in different plants. This may also be a coincidence that the reduced volatiles (e.g. dominant sesquiterpenes or aromatic compounds) in maize act as repellents which can mask the attractive signals^27^.

Given the above results, we conclude that laminarin evidently influences tea defense responses and consequently alters the interactions of the plant with other organisms. This notion was also confirmed in field experiments where laminarin-treated plants attracted lower numbers of TLH adults and nymph, which resulted in less egg on the treated plants (Fig. 8a–d). Parasitism by *S. empoascae* was augmented on the laminarin-treated plants (Fig. 8e). The field experiments indicated that laminarin holds a control potential and can be thereby used to reduce pest damage in tea fields.

In conclusion, we presented evidences supporting that laminarin can enhance tea plants’ direct and indirect resistance to TLH, by controlling the MAPK signaling, H_2_O_2_, SA, and ABA and other defense-related chemicals. This research provided a new strategy for enhancing the integrated management of a harmful tea sucking-insect by improving defense-related signaling transduction mechanisms. Next, the possible effects of chitinase, PAL, and callose on the tea plant’s defense against TLH require further investigation. The three signal molecules SA, ABA, and H_2_O_2_, merit further attention as a potential modulator of the tea plant’s defenses against TLH.

## Material and Methods

### Plant and insects

The widely cultivated tea (*C. sinensis* L.) cultivar ‘Longjing 43’ was used in the current experiments. *E. onukii* nymphs and *Stethynium empoascae* Subba Rao colonies were collected from tea plants at the Tea Research Institute of the Chinese Academy of Agricultural Sciences (TRICAAS), Hangzhou, China. Mated 2-day-old *E. onukii* adults were subjected to oviposition and preference assay experiments. Only females *S. empoascae* were tested in the olfactometer bioassay. The tea plant and insects used in this study were prepared as described previously^19,55^.

### Plant treatments

#### Laminarin treatment

Laminarin from *L. digitata* (Sigma-Aldrich) was prepared at a concentration of 200 mg L^−1^ in distilled water with 0.01% Tween-20 and applied to both the upper and lower tea leaf surfaces by using a handheld sprayer. Control plants were sprayed with water containing 0.01% Tween-20.

#### TLH treatment

For volatile collection, individual tea plants covered with square net cages (65 cm × 65 cm × 65 cm) were infested with 150 TLH adults. Noninfested tea plants covered with square net cages were set as control plants (C).

### JA, SA, ABA and H_2_O_2_ analysis

The second fully expanded leaves of control (Con) and laminarin-treated (Lam) tea plants were used to quantify plant signaling molecules. The samples with five repeats were harvested at 0, 1, 2, 4, 8, 12, 24 and 48 h after treatment. For signaling molecules analysis, 50 mg of freeze-ground tea sample and 50 mg of polyvinylpolypyrrolidine (PVPP) were used to homogenize in 1 mL 0.2 mol L^−1^ sodium phosphate buffer (SPB, titrated with 1 M citric acid). The homogenate was centrifuged at 12,000 g for 10 min at 4 °C, and the supernatants were used for chemical analysis. The method used for JA, SA, and ABA analysis was based on the study of Lou and Baldwin^56^. JA, SA, and ABA were analyzed by high-performance liquid chromatography–tandem mass spectrometry (HPLC-MS/MS) with ^13^C_2_-JA, D_4_-SA, and D_6_-ABA as the internal standards. For H_2_O_2_ analysis, the homogenized samples were individually mixed with 1 mL of deionized water, and the supernatants were collected by microcentrifugation (12, 000 g) of the extract at 4 °C for 10 min. H_2_O_2_ concentrations were then determined as described by Lou and Baldwin^57^.

### Localization of H_2_O_2_

For detection of the accumulation of H_2_O_2_, laminarin-treated tea leaves or control leaves were inserted into 0.1% fresh 3, 3′-diaminobenzidine (DAB) solution (pH 3.8) prepared in 50 mM Tris-HCl buffer (pH 7.8) and incubated in light until brown spots were observed (5 to 6 h). After staining, the background was bleached in 95% ethanol in a boiling water bath, and then photographed.

### Quantitative real-time PCR (qRT-PCR)

The second fully expanded leave samples with five repeats were harvested from control (Con) and laminarin-treated (Lam) tea plants at different time points. Total RNA isolation, reverse transcribing, and qRT-PCR assay were following the method as described by Xin *et al*.^55^. The qRT-PCR program included of a preliminary step at 95 °C for 30 s, followed by 40 cycles of a denaturation at 95 °C for 10 s, and an annealing and extension step at 58 °C for 30 s. The primers used for transcript analysis have been listed in Supplementary Table S1.

### Production of specific monoclonal antibodies (mAbs) and immunoblot analyses

CsMAPK-, CsOPR3-, CsWRKY3-specifc monoclonal antibodies (mAbs) were produced and purified according to the method described by Zhang *et al*.^58^. Total protein of tea leave was extracted via Plant Total Protein Extraction Kit (Sigma-Aldrich), following the manufacturer’s instructions, and the extraction procedure was modified as follows: 50 mg of tea leaf powder was used to homogenize in the extraction buffer, and 50 mg of PVPP was added to remove phenolic compounds from the leaf samples. The protein samples were separated by SDS-PAGE and blotted onto PVDF membranes for immunoblot analysis.

### Defense enzyme activity assay

The activities of different defense enzymes in the control (Con) and laminarin-treated (Lam) tea leaves were measured 0, 0.5, 1, 2, 3, 4, 5, 6, 7 day after treatment, with five independent biological samples. The sample (50 mg) was homogenized in 1 mL 0.2 mol L^−1^ SPB solution of pH 5.6 consisting of 50 mg PVPP. The homogenate was centrifuged at 12,000 g for 15 min at 4 °C, and the supernatants were used for analysis. PPOs activity was estimated by following the method of Xin *et al*.^55^. PAL activity was estimated following the method of Chandra *et al*.^59^. The conversion from L-phenylalanine to transcinnamic acid was employed to determine the PAL activity in the control and treated leaves. The enzyme activity was showed as the synthesis of transcinnamic acid (n mol) min^−1^ g^−1^ protein. Chitinase activity was determined by measuring the reducing end group N-acetamino-glucose produced from colloidal chitin^60^. One unit of chitinase activity was defined as the amount of enzyme that liberates 1 μg of N-acetamino-glucose per min at pH 5.4 and 50 °C.

### Callose assay

The treatments and sampling methods were similar as those for the defense enzyme activity assay (described above). The extracting and testing of callose contents in tea leaves were evaluated by the methods described by Liu *et al*.^12^. Optical density values were detected with the Cary Eclipse fluorospectrophotometer (Varian Co., Palo Alto, CA). The callose contents of tea leaves were then calculated in terms of the fresh weight. Callose staining assay was evaluated following the method of Zhang *et al*.^61^. Leaves were stained with 0.01% aniline blue and examined with a Zeiss Axiophot D-7082 fluorescence microscope with an excitation filter of 365 ± 25 nm, a 400 nm dichroic mirror, and a 450 nm longpass emission filter.

### Collection, isolation and identification of tea volatiles

Tea volatiles from the control plants (Con), plants treated with laminarin for 48 h (Lam), plants infested by TLH (150 insects per tea plant) for 24 h (TLH), and the plants treated with laminarin for 24 h and followed infestation by TLH (150 insects per tea plant) for 24 h (Lam + TLH) were collected. The tea volatiles were isolated and identified using the method described by Xin *et al*.^55^. Each treatment was replicated six times.

### TLH preference measurement

To assess the preference of TLH adults on control and laminarin-treated plants, two tea branches (one control plant *vs*. one laminarin-treated plant) with similar growth shape were covered in a glass cylinder, into which 15 gravid adult TLH females were released. The number of TLH on each branch was recorded 0.5, 1, 2, 4, 8, 12, 24, and 48 h after the release of the TLH. After 72 h, TLHs were removed, and the eggs on each plant were counted. Six times were replicated. The survival rates of TLH nymphs were also detected. Pots with one plant (control or laminarin-treated plants) were individually confined with square plastic cages into which 50 newly hatched TLH nymphs were introduced. Three days after the experiment, the number of alive TLH nymphs on each plant was counted. Each treatment was repeated six times.

### Olfactometer bioassay

The responses of *S. empoascae* female adults to tea volatiles were measured in a Y-tube olfactometer through the method described by Xin *et al*.^58^. The four different treatments were used: control (Con); Lam; TLH; and Lam + TLH. These treatments were the same as those for the collection and isolation of tea volatiles (described above). *S. empoascae* females were then allowed to choose between plants Con vs. Lam or TLH vs. Lam + TLH. Six times were replicated in this experiment.

### Field experiment

Field experiments were carried out in October 2017 in Hangzhou, China. The experimental field was divided into 9 plots (10 m × 10 m), and was randomly assigned to three control plots, three laminarin-treated plots, and three blank plots. Each plot surrounded by an isolation belt with a width of 1.5 m (Fig. S3). Plots were sprayed with 5 L of 200 mg L^−1^ laminarin solutions containing 0.01% Tween-20 (Lam), 5 L water with 0.01% Tween-20 (Con), and applied to leaf surfaces using a handheld sprayer. Nonmanipulated plots were used as blanks (Blk). The number of TLH nymphs, female and male adults that were present in different plots was investigated 0, 5 and 10 d after the treatments. Ten days after the treatments, 20 tea branches from each plot were cut off and brought to the laboratory to dissect and record parasitized and nonparasitized TLH eggs with a microscope.

### Data analysis

All tests were carried out with Statistica (SAS Institute, http://www.statsoft.com). Differences in data of field analysis and tea volatile levels were analyzed via analysing variance followed by Duncan’s multiple-range test. Student’s *t*-test was used for comparing two treatments. For the olfactometer test, the differences between the numbers of *S. empoascae* entering each arm of the olfactometer (a response equal to 50:50) for each paired treatment were analyzed by the Kruskal-Wallis test (χ^2^ approximation).

## Supplementary information

Dataset 1
