# Supplementary material for: A Disease Resistance Elicitor Laminarin Enhances Tea Defense against a Piercing Herbivore Empoasca (Matsumurasca) onukii Matsuda
Source: Sci Rep. 2019 Jan 28;9:814. doi: 10.1038/s41598-018-37424-7 (PMC6349920; doi:10.1038/s41598-018-37424-7)

A Disease Resistance Elicitor Laminarin Enhances Tea Defense against a Piercing Herbivore *Empoasca* (*Matsumurasca*) *onukii* Matsuda

Zhaojun Xin, Xiaoming Cai, Shenglong Chen, Zongxiu Luo, Lei Bian, Zhaoqun Li, Lingang Ge, Zongmao Chen

Supplementary Table S1: Description of the primers used for qRT-PCR.

Gene	Forward Primers (5′-3′)	Reverse Primer (5′-3′)
CsMAPK	AGGTGAGTTGATTGGTTGCG	CCGCAATTGAAACCTGCTTG
CsWRKY3	CACCACCCACCACTCACA	AAAACCTTCATACCTCCT
CsPPO	GGTGGATCGAGACGTGTTTG	TCATCCTCAGCTCCCAACTC
CsFLS1	GCTCACACAGACATGTCCAC	ACACAGCCCTGTACTTTCCA
CsCHIT1	GAGGCCCAATCCAACTTTCC	AAGGCTTCGGGTATTGTGGA
CsCalS	CCTGAGCTCTTCGACAGTGA	AAGCATGAAAGCGGCAAAGA
GADPH	ATACCACGTCATCCTCGGT	ACTTATGATGAAATCAAAGCTGC

Supplementary Fig. S1: Effects of laminarin treatment on the transcript and protein levels of CsOPR3. (a) Mean transcript levels (+ SE, n = 5) of *CsOPR3* in leaves of tea plants treated with laminarin at a concentration of 200 mg L^{-1} (Lam), relative to controls (Con). (b) Western blot analysis of the accumulation of CsOPR3. The grouping of blots was cropped from different parts of the same gel.

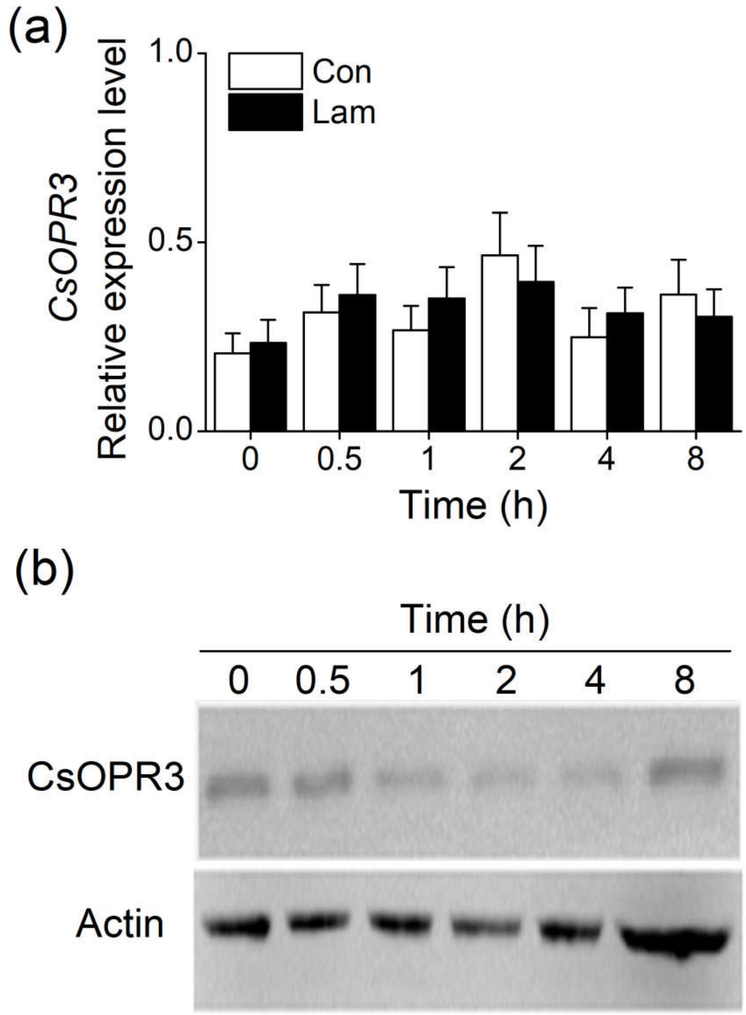

Supplementary Fig. S2: Relative changes in spider abundance (+SE, n = 3) in the field 5 and 10 days after tea plants were sprayed with laminarin at a concentration of 200 mg L^{-1} (Lam).

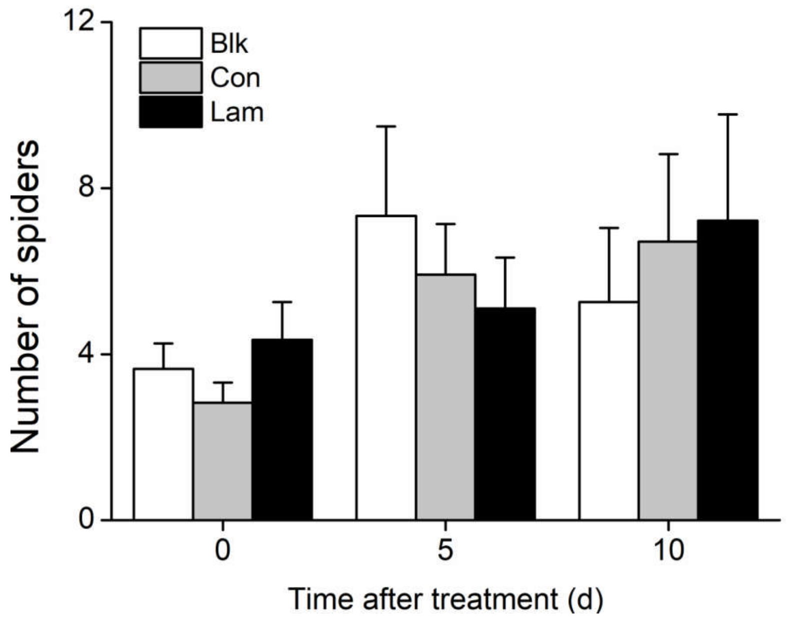

Supplementary Fig. S3: Design of the field experiment. Blk, plants were non-manipulated; Con, plant were sprayed with water with 0.01% Tween-20; Lam, plants were sprayed with 5 L of 200 mg L^{-1} laminarin solutions containing 0.01% Tween-20

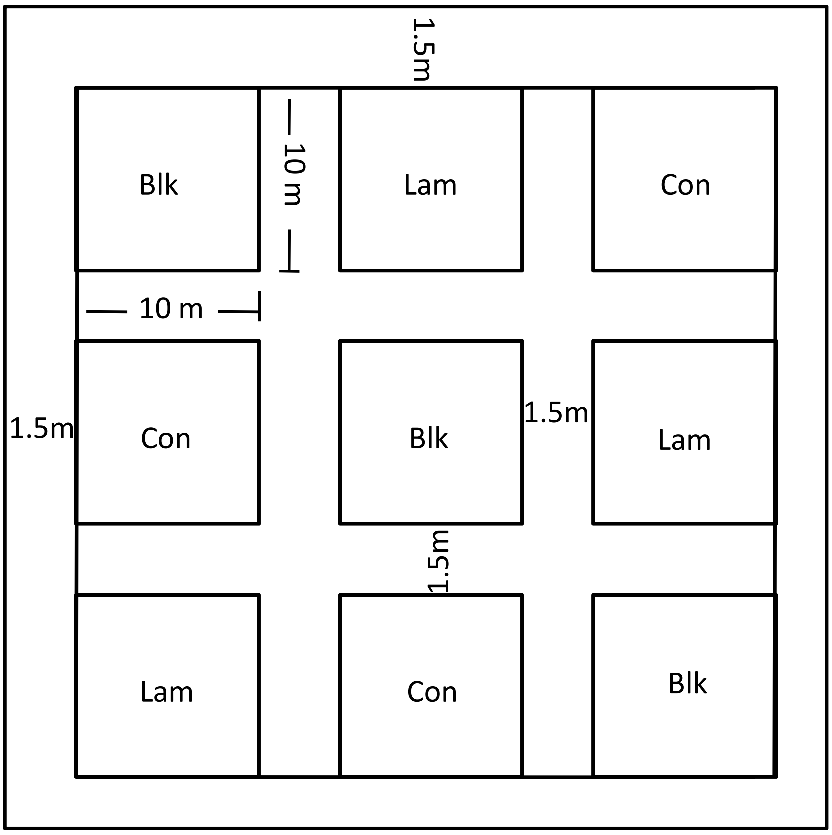

Supplement: Supplementary file 1 — Dataset 1 [file 41598_2018_37424_MOESM1_ESM.pdf]
